# Spectrally efficient optical orthogonal frequency division multiplexing

**DOI:** 10.1098/rsta.2019.0180

**Published:** 2020-03-02

**Authors:** Arthur James Lowery

**Affiliations:** Department of Electrical and Computer Systems Engineering, Monash University, Clayton, Victoria 3800, Australia

**Keywords:** optical communications, spectral efficiency, orthogonal frequency division multiplexing, intensity modulation, direct detection

## Abstract

This paper charts the development of spectrally efficient forms of optical orthogonal frequency division multiplexing (OFDM) that are suited for intensity-modulated direct detection systems, such as wireless optical communications. The journey begins with systems using a DC-bias to ensure that no parts of the signal that modulates the optical source are negative in value, as negative optical intensity is unphysical. As the DC-part of the optical signal carries no information, it is wasteful in energy; thus asymmetrically clipped optical OFDM was developed, removing any negative-going peaks below the mean. Unfortunately, the clipping causes second-order distortion and intermodulation, so some subcarriers appear to be unusable, halving spectral efficiency; this is similar for unipolar and flipped optical OFDM. Thus, a considerable effort has been made to regain spectral efficiency, using layered techniques where the clipping distortion is mostly cancelled at the receiver, from a knowledge of one unpolluted layer, enabling one or more extra ‘layers/paths/depths’ to be received on the previously unusable subcarriers. Importantly, for a given optical power and high-order modulation, layered methods offer the best spectral efficiencies and need the lowest signal-to-noise ratios, especially if diversity combining is used. Thus, they could be important for high-bandwidth optical fibre systems. Efficient methods of generating all layers simultaneously, using fast Fourier transforms with their partial calculations extracted, are discussed, as are experimental demonstrations in both wireless and short-haul communications links. A musical analogy is also provided, which may point to how orchestral and rock music is deciphered in the brain.

This article is part of the theme issue ‘Optical wireless communication’.

## 1. Introduction

Communications systems have many metrics to quantify their performance, for example their data rate (bit s^−1^) and transmission distance are key functional specifications. Added to these are power consumption, reliability/availability, security and the ability to work with other systems without interference. Mobile systems, including some optical wireless, also need to consider coverage; that is, over what area will the link perform?

**Figure 1.**
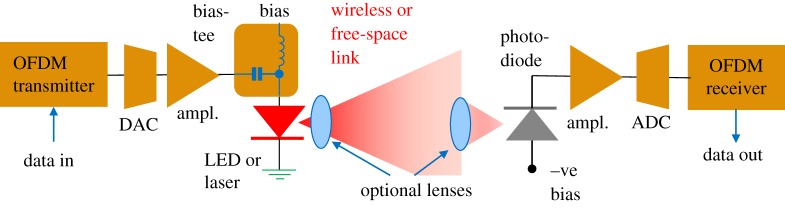
Block diagram of a typical wireless (or free-space) optical link. The use of lenses increases the received power substantially but reduces the area of transmitter coverage, or imposes directionality on the receiver. (Online version in colour.)

This paper is, however, about spectral efficiency, which has become important in optical fibre communications as it indicates the ability to pack more wavelength-multiplexed channels into a single optical fibre [1]. However, it is also a key parameter in wireless (fibre-less) optical communications, as illustrated in figure 1, and single-wavelength systems that are used for short-range interconnects. This is because each signalling rate of each wavelength of most optical systems is ultimately limited by the electrical bandwidths of their optoelectronic components, such as light-emitting diodes, lasers, optical modulators and photodiodes and of the associated electronics, including analogue-to-digital and digital-to-analogue converters [2,3]. Thus, this paper will hereon in refer to electrical spectral efficiency, defined as the bit rate per unit electrical bandwidth (bit s^−1^ Hz^−1^). This efficiency can be increased by coding many bits per symbol, such as by using higher order quadrature amplitude modulation (QAM) [4]; for example, 16-QAM will carry log_2_(16) = 4 bits symbol^−1^. Using higher order QAM, say up to 1024 QAM, increases spectral efficiency: it also increases the required signal-to-noise ratio (SNR) at the receiver substantially. Thus, there is a trade-off between transmission distance and spectral efficiency for a given transmitted power.

In optical wireless systems [5], and many fibre systems, there is also a limit on total optical power. In the early days, this was usually due to the falling reliability of the laser or LED at higher powers. More recently, this is due to safety standards, which are particularly stringent at visible wavelengths as visible light is naturally focused onto the retina creating extremely high power densities at the eyes' photoreceptors. Thus, this paper will concentrate on the electrical spectral efficiencies available for a given optical power, for a number of signalling schemes.

The paper is organized as follows. Firstly, optical orthogonal frequency division multiplexing (OFDM) schemes will be introduced that reduce the unmodulated optical power, but lower the spectral efficiency. Secondly, two- then three-layer schemes for regaining the spectral efficiency will be discussed. Thirdly, the cost of increasing the spectral efficiency, in terms of the required electrical SNR at the receiver, will be compared for various schemes all operating with the same optical power; this section shows the advantage of layered schemes for spectral efficiencies above 3 bit s^−1^ Hz^−1^. Experimental demonstrations, both in wireless and fibre systems, will be compared. The layered schemes require significantly more signal processing than conventional transceivers; some methods of reducing this will be presented. Finally, other improvements will be discussed before a conclusion is presented.

## 2. Optical orthogonal frequency division multiplexing

OFDM uses multiple subcarriers to transmit a given data channel [6]. The frequencies and signalling rates of these subcarriers are arranged so that a receiver can separate them without mutual interference. The simplest method is to arrange the frequency spacing to equal the inverse of the duration of a raw OFDM symbol, which makes all subcarriers periodic within the symbol; thus, they can be perfectly separated using a Fourier transform (FT). By ‘raw’, the OFDM symbol does not include cyclic extensions to the OFDM symbol, which are used in dispersive channels, but are discarded before the FT. A detailed history of the early ‘radio’ implementations of OFDM can be found in [7].

Early patents disclosed that OFDM could work over any frequency range of subcarriers, even optical, though all-optical systems were not demonstrated until 2002 with the FT being implemented by an optical interferometer [8]. More conventionally, an intensity-modulated direct detection (IM-DD) system was shown to be able to carry OFDM subcarriers in 1996, with an increased resistance to impulsive noise [9]. Similarly, hybrid-fibre coax systems [10] distribute CATV signals, including data on subcarriers (data over cable service interface specification—DOCSIS [11]). The key to these systems is to add a high DC-bias to the electrical signal before it modulates the laser, so that the bipolar electrical signal modulates the light source above its lowest light level; that is, there is always positive light intensity. This is equivalent to limiting the optical modulation depth [10]. The bias has to be strong because OFDM signals have large positive and negative peaks in their waveform, due to the phases of the subcarriers occasionally aligning (depending on the data being sent).

The problem with adding a high bias is that it substantially increases the mean optical intensity, especially if infrequent ‘clipping’ of the negative peaks is to be avoided. Any clipping adds to the noise of the demultiplexed subcarriers and also reduces their amplitudes. This degradation is especially troublesome for high-order QAM, where high-quality signals are required to enable the separation (e.g. by thresholding, also known as slicing) of the received signal into sets of data bits without error. A high mean optical intensity may cause reliability issues with the laser, or safety issues, for example.

**Figure 2.**
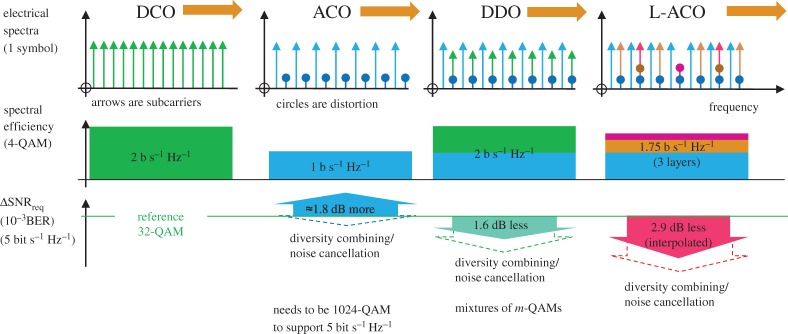
Evolution of optical OFDM systems from DC-biased (DCO), through asymmetrically clipped (ACO), then symmetrically clipped and DC offset (ADO) to layered/enhanced (LACO). The top row is the electrical spectra for one-symbol; the middle row is the relative spectral efficiency for 4-QAM constellations, and the bottom row shows the required electrical SNR relative to DCO-OFDM for 5 bit s^−1^ Hz^−1^ spectral efficiencies. (Online version in colour.)

In 1996, Carruthers & Kahn [5] suggested methods to reduce the bias overhead for multi-carrier modulation by clipping each subcarrier separately, then You & Kahn [12] proposed block coding or variable bias in 2001; these required 3–7 dB more power than on–off keying (OOK). In 2005, Lowery & Armstrong [13] and Armstrong & Lowery [14] provided two methods based on clipping the sum of the subcarriers, rather than individual subcarriers; simulations showed an advantage over OOK; both methods used clipping at exactly the mean level to remove excursions below the mean level, and both also sacrificed some of the subcarriers, to accommodate the substantial clipping-induced distortion, which was mostly second-order harmonic and intermodulation distortion. Simulations identified that clipping exactly at the mean level was optimal, in terms of the received signal quality for a given optical power. In [13], the signal distortion caused by clipping mostly falls within a spectral gap with a bandwidth equal in the signal bandwidth. In [14], only odd subcarriers are generated, and clipping of these created distortion only at the frequencies of the (deleted) even subcarriers, as illustrated in figure 2. Thus, the distortion could be completely rejected by the FT at the receiver. Both methods are known as ‘asymmetrically clipped optical (ACO)-OFDM’, though the latter is subject of most research for wireless systems. The ‘frequency gap’ idea is more suited to optical fibre systems using modulation of the optical field rather than intensity (to enable electronic compensation of chromatic dispersion in the fibre dispersion [15]), where it is used to reject distortion caused by direct detection of these field signals that leads to unwanted frequency-difference products [16].

Both ACO-OFDM solutions rely on ‘throwing away’ half of the subcarriers, which means that the electrical bandwidths of the transmitter and receiver need to be doubled for the same data. Similar methods, such as those relying on sending the negative-valued portions of the signal in a second frame (Flip-OFDM [17–19], unipolar OFDM [20]) also suffer from halved spectral efficiency. This has led to intense research on how to reclaim this bandwidth, for both the gapped and odd subcarrier methods, and also for Flip and Unipolar OFDM.

The gapped method has proven more useful for fibre systems using optical field rather than intensity modulation because when used in combination with single-sideband modulation with carrier suppression, OFDM's single-tap equalization can be used to compensate chromatic dispersion, even with a single-photodiode receiver [15]. The photodiode's square-law detection causes an unwanted *signal × signal* beat product, but this fortuitously falls within the gap. Just as with intensity-modulated systems, regaining spectral efficiency has been a major research effort in field-modulated systems [21]; Kramers–Kronig receivers provided an interesting solution based on the relationship between phase and amplitude of minimum-phase waveforms [22]. Interestingly, clipping of the received waveforms appears to be able to improve these systems [23].

## 3. Regaining spectral efficiency: two-layer schemes

The key to spectral reclamation techniques is that clipping distortion is deterministic, and so it should be possible to calculate it from the received signal, and then subtract it to ‘clean-up’ the unused portions of the spectrum, so that new subcarriers can be revealed. This also works for Flip/U-OFDM signalling, but is due to inherent symmetries in the time domain signals.

### (a) Asymmetrically clipped and DC-biased optical orthogonal frequency division multiplexing

**Figure 3.**
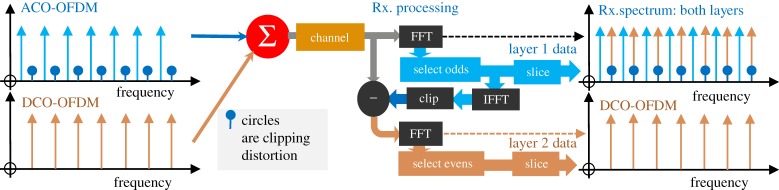
Processing of ADO-OFDM to clean the DCO-OFDM of ACO-OFDM interference. The select odds block separates out the ACO-OFDM signal in the frequency domain. This is then clipped to recreate the distortion that fell on the evens at the transmitter. This distortion is subtracted from the received signal to reveal the DCO-OFDM signal. (Online version in colour.)

In 2011, Dissanayake *et al.* [24] proposed cancellation of clipping distortion and added DC-biased optical OFDM (DCO-OFDM) subcarriers to all of the newly available ‘even’ subcarrier slots. As illustrated in figure 3, the DCO-OFDM subcarriers were used because they create no distortion themselves (they are not clipped before adding to the already clipped ACO-OFDM subcarriers). Later work showed an advantage in electrical SNR for a given optical power over conventional techniques for SEs above 3 bit s^−1^ Hz^−1^ [25]. At the receiver, the ACO-OFDM subcarriers were isolated from the spectrum using an FT, then re-clipped to recreate the transmitted ACO-OFDM signal, which was subtracted from the received signal to reveal the DCO-OFDM subcarriers. No slicing nor thresholding was used during the recreation process; thus, noise from the ACO-OFDM spectrum would degrade the DCO-OFDM channels.

### (b) Hybrid asymmetrically clipped optical–orthogonal frequency division multiplexing

This 2014 method by Ranjha & Kavehrad uses QAM-modulated ACO-OFDM on the odd subcarriers and clipped pulse-amplitude modulation discrete multi-tone (PAM-DMT) on the even subcarriers [26] to achieve various spectral efficiencies depending on the mix of constellation sizes and apportionment of power. As with ADO-OFDM, but with thresholding, the ACO-OFDM waveform is recreated at the receiver and subtracted to reveal the PAM-DMT subcarriers. Interestingly, if PAM-DMT subcarriers are clipped, the resultant distortion only falls on their own (even) frequencies, so does not pollute the (odd) ACO-OFDM subcarriers; the distortion falls in quadrature with the desired PAM-DMT signals, so can be discarded. A fundamental disadvantage of PAM is that it requires higher SNRs than QAM for a given bits symbol^−1^, because it only uses one signal dimension and each additional bit requires a doubling of the number of levels and approximately a doubling of power; this means that this system is optimal when the PAM subcarriers transport fewer data than the QAM subcarriers.

### (c) Asymmetrically and symmetrically clipping optical orthogonal frequency division multiplexing

This 2015 scheme by Wu & Bar-Ness uses ACO-OFDM on the odd subcarriers and a Flip/U-OFDM-type signal on the even subcarriers at only one-half of the baud rate of the ACO-OFDM signal [27]. It follows from a 2012 paper improving the error rate performance of ACO-OFDM [28]. Using mixes of constellation sizes on the two layers enables various spectral efficiencies and generally requires lower optical powers than ADO-OFDM, because no bias is required on either layer. Thresholding/slicing was not used when cancelling the ACO-ODFM clipping distortion.

## 4. Regaining spectral efficiency: multi-layer schemes

In some techniques, more than two layers share the data load, and multiple stages of cancellation are used at the receiver, to ‘peel-away’ the layers, like an onion. In these schemes, the first layer must be unpolluted by the deeper layers, as it has to be decoded and reconstructed so that its distortion can be removed from deeper layers. The sharing of data is usually performed with 50% of the data of the equivalent DCO-OFDM on Layer 1, 25% on Layer 2, 12.5% on Layer 3, etc., which gives diminishing returns in spectral efficiency, at a cost of more processing every time a layer is added. The advantage of using multiple layers is that all layers can use power-efficient modulation, such as QAM with clipping; this leads to the lowest SNR requirements of all schemes.

### (a) Enhanced unipolar orthogonal frequency division multiplexing

**Figure 4.**
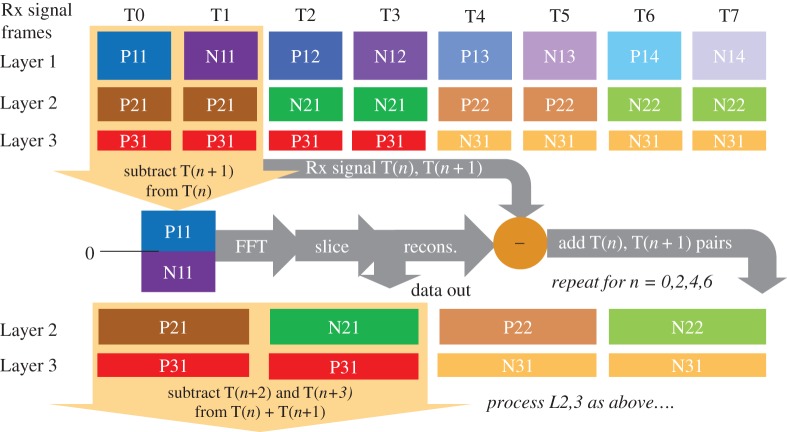
Processing of EU-OFDM frames T to extract layers. P indicates a sub-frame carrying the positive parts of the signal; N indicates a frame carrying the inverted negative parts (so they are now positive). The fact that the frames of deeper layers are repeated allows the deeper layers to be discarded by time domain subtraction of adjacent frames, and also a bipolar signal; e.g. subtracting frame T1 from frame T0 provides Layer 1's bipolar signal P11-N11. (Online version in colour.)

Enhanced unipolar orthogonal frequency division multiplexing (EU-OFDM) [29] uses the superposition of time domain signals at various layers (called ‘depths’), where each U-OFDM symbol (*positive* frame, P, followed by a *negative* frame, N, like Flip-OFDM) in the deeper layers is transmitted more than once as a simple repeat. This is illustrated in figure 4. Obviously, the data rate of each deeper layer is halved due to the repetition, and the effect of the repetition on its subcarriers is to narrow their spectra. Experimental results showed a 2-dB electrical drive power advantage for 16-QAM over DCO-OFDM for a BER = 10^−3^ at 80 Mbit s^−1^ [30]. The repetition of the deeper layers allows them to be cancelled when a higher layer is decoded. Consider the initial decoding of Layer 1, shown in figure 4. By subtracting pairs of frames, say T1 from T0, a bipolar frame for Layer 1 will be revealed as it equals P11-N11. The adjacent frames of deeper layers at this time cancel, e.g. P21–P21 = 0 and P31–P31 = 0. Thus, Layer 1 can be reconstructed without interference from deeper layers. The reconstructed signal can be clipped, and then subtracted from the complete set T0–T7, to reveal Layers 2 and 3. Next frames T3 and T4 can be subtracted from T0 and T1, to obtain a bipolar signal for Layer 2, while also cancelling Layer 3.

EU-OFDM places the subcarriers of all of the layers at the same frequencies (albeit the higher layers have narrower sinc-shaped spectra), which unfortunately means that any errors when slicing the constellations of the higher layers results in strong error vectors at the deeper layers, causing a cascade of bit errors. The optical and electrical spectra are not flat, because some frequencies support multiple layers. This may reduce the performance of the system. A further issue with Flip/U-OFDM is that the adjacent positive and negative signal blocks must not interfere with one another in a bandwidth-limited channel. This either requires time domain equalization before the processing of the blocks or additional cyclic prefixes between all frames.

### (b) Generalized EnhaNcEd unipolaR orthogonal frequency division multiplexing

Islim *et al*. have proposed that the spectral efficiency of layered schemes can be made to exactly match that of DCO-OFDM simply by using different constellation sizes and transmit powers on different layers [31]. The electrical SNR requirement was about 4 dB better than DCO-OFDM for a BER of 10^−3^.

### (c) Layered/enhanced ACO-OFDM (LACO-OFDM or L/E-ACO-OFDM)

**Figure 5.**
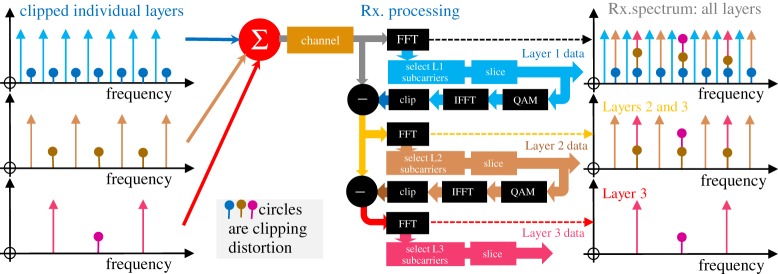
Processing of LACO-OFDM to extract layers. Layer 1's subcarriers are selected after the top-right FFT and after slicing to extract data bits, and the clipped waveform of Layer 1 is reconstructed by a QAM modulator, inverse FFT (IFFT) and clipper. This waveform is subtracted from the received signal to reveal Layers 2 and 3. Note that the reconstruction includes slicing (hard decisions), to prevent noise from Layer 1 being passed to Layer 2's processing (though hard errors would be propagated). (Online version in colour.)

As shown in figure 5, LACO-OFDM successively fills the frequency slots with layer upon layer of subcarriers. Unlike EU-OFDM, no slot carries more than one subcarrier. Layer 1 is conventional ACO-OFDM, occupying only the odd frequency subcarriers (2*n *+ 1); *n* integer including zero. Layer 2 fills the even frequencies that are 2(2*n *+ 1), for example 2, 6 and 10. Layer *m* fills 2*^m^*^−1^(2*n *+ 1). At the receiver, Layer 1 can be decoded as in conventional ACO-OFDM, because it has no interference from higher layers. A facsimile of Layer 1's transmitted signal can be constructed and subtracted from the received signal, to reveal Layer 2 free of Layer 1's distortion. Importantly no clipping distortion from Layer (*n *+ 1) falls on Layer *n*, so Layer *n* can be decoded without error if the reconstruction of Layer (*n* − 1) is error free.

Layered/enhanced ACO-OFDM has been invented independently by several groups, via various routes. In 2010, Chen *et al.* [32] published a successive decoding method at a conference, which formed a small part of a later journal paper [33] and seems to have been missed by many groups. In March 2015, a PCT patent application was published on EU-OFDM (Inventors: Tsonev and Haas) but noting that ACO-OFDM could be used as a basis for the frames [34]. In May 2015, Wang *et al.* [35] presented numerical results from the layered ACO-OFDM, and in December 2015, Islim *et al.* [36] published the PCT framed system using ACO-OFDM; the repetition of frames in the higher layers causes the subcarriers of all layers to fall at the same frequencies, albeit with narrower spectra. In October 2015, Lowery published a description based on musical chords (each of odd harmonics of a fundamental) at octave intervals [37], each describing a layer, and showed that the method offered the best sensitivities over other methods for higher order QAM modulations. In contrast with Islim *et al.*, each frequency slot was used only once, if at all. In 2017, Mohammed *et al.* [38] added diversity combining to layered ACO-OFDM and reached similar conclusions in performance comparisons. Diversity combining uses the information in the clipping distortion as part of the recovery process and has been shown to improve errors rates in both clipped [39,40] and flipped [17] OFDM systems.

Errors in Layer *n* may not necessarily propagate to Layer (*n *+ 1), because the error vectors are small, as they are residues of the un-cancelled clipping distortion and their power is distributed across many subcarriers. Inter-layer error correction reduces error propagation still further, as proposed by Hanzo's group [41,42] and also disclosed by Song's PhD [43].

### (d) Spectral and energy efficient orthogonal frequency division multiplexing

This 2014 scheme requires only one FT at the receiver, where the layers are called Paths [44]. The top path is ACO-OFDM, and the lower layers are combinations of ACO-OFDM with repeated frames that are flipped versions of the first frames. Unlike EU-OFDM, all layers have the same OFDM symbol duration, which is the reason that only a single FT is required––an obvious advantage. Slicing upon decoding was not used initially, but later versions use slicing and ACO-OFDM on all layers [45]; thus, these require multiple FTs during the decoding process, as with other layered ACO-OFDM methods. Using ACO-OFDM ensures that all of the subcarriers are periodic within a common symbol duration, so advantageously share one common cyclic prefix per symbol, thus lowering the CP overhead compared with EU-OFDM. At the receiver, the channels are successively decoded, starting with the original odd subcarrier channel. Thus, for reasonable SNRs, the penalty due to imperfect cancellation of a channel is very small, as there is little noise on the cancellation waveform.

### (e) Spectrally enhanced amplitude modulation discrete multi-tone and augmented spectral efficiency discrete multi-tone

In 2015, Islim *et al.* [46] extended generalized EnhaNcEd unipolaR orthogonal frequency division multiplexing (GREENER-OFDM) to enable multiple streams of PAM-DMT to be transmitted. This used a frame structure similar to Flip-OFDM, with cyclic prefixes between each frame, and considered optimum constellation sizes for each stream. A simplified frequency domain generation method, similar to used in LACO, was then published which uses a FT per ‘depth’ [47]. The idea in both papers is that the clipping distortion of a PAM subcarrier on the quadrature (sine) component of a complex signal will fall on the in-phase (cosine) component only. If this is used for Depth/Layer 1, then the receiver is able to detect its quadrature component, reconstruct the in-phase clipping distortion and then subtract it from the received signal. Thus, the in-phase components can now also be used for signalling, providing that their distortion products do not fall on the quadrature component. Fortunately, all clipping distortion of in-phase signals falls upon them in-phase, so in the second paper, a similar arrangement to LACO-OFDM can be used to ‘fill-up’ the in-phase subcarriers, starting from the odd subcarriers for Depth 2.

## 5. Signal-to-noise ratio cost of increasing spectral efficiency

The most common way of gaining spectral efficiency in communications systems is to use higher-order quadrature amplitude modulation formats, denoted *m*-QAM, where *m* is the number of distinct symbols in a two-dimensional constellation. The number of bits required to specify the position of a symbol is log_2_(*m*); for example, 256-QAM will carry eight bits of data for each transmitted constellation pattern. If *m* is increased, then the separation of the constellation points for a given transmitted power will decrease, making the impact of noise more likely to misplace a constellation point and so cause a symbol error, which usually produces a bit error if the constellation is Gray coded. Alternatively put, for a given noise level, higher-order QAM constellations require more power.

**Figure 6.**
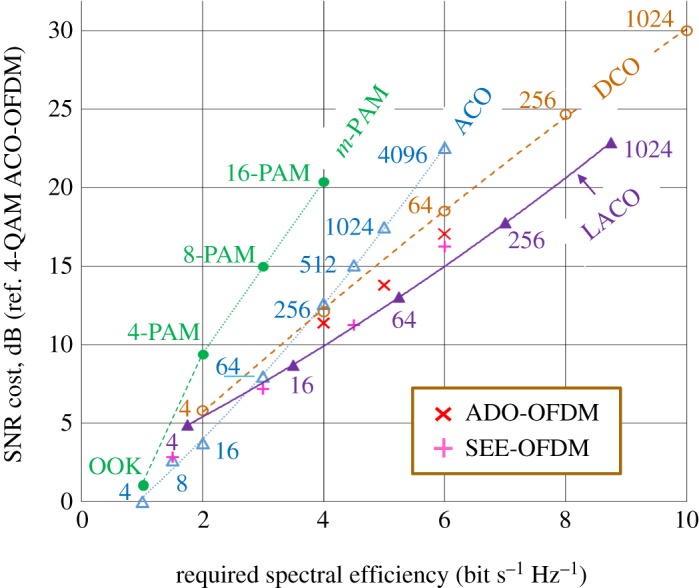
Comparisons of the costs (in electrical SNR) of increasing the spectral efficiency using various optical OFDM schemes, with various constellation sizes. Note that results for noise cancellation or diversity combining are not shown, and these can give around 2 dB further improvements for ACO and LACO. (Adapted with permission from [48]). (Online version in colour.)

Figure 6 compares the ‘cost’ of increasing the spectral efficiency of various OFDM schemes, each for a number of *m*-QAMs [48]. The cost is defined as the increase in *electrical* SNR required to support an increase over the reference system, which is 4-QAM ACO-OFDM. In every case, the received mean optical power is set to be the same. Also included is PAM, where only one Cartesian dimension is used; note that the actual bandwidth of PAM depends on the pulse shaping used, and in the Nyquist limit (using sinc-shaped pulses), PAM could be twice as spectrally efficient as shown; detailed comparisons for a range of modulator bandwidths are given in the papers of Sharif *et al.* [2] and Perin *et al.* [3]. In these PAM simulations, the transmitted pulses were unshaped, and the electrical receiver noise was band-limited by a fourth-order Bessel filter with a bandwidth of 70% of the data rate. For IM-DD systems dominated by the thermal noise of the receiver (typically systems without optical amplification), the electrical SNR will improve 2 dB for each 1 dB increase in received optical power, due to the photodiode's square-law detection (IM/DD), where photocurrent is proportional to optical power. Thus, the ‘cost’ of increased spectral efficiency, *in terms of optical power* (dB), is half that shown by figure 6. Although ACO-OFDM provides the lowest SNR cost at low spectral efficiencies, its SNR cost rapidly increases with spectral efficiency. This is because ACO-OFDM requires a constellation size of *m*-squared compared with schemes that do not have a loss of half the subcarriers, e.g. 1024 constellation points rather than 32 for schemes with full spectral efficiency. This fact makes layered schemes very attractive for high spectral efficiencies because the extra data are carried by additional layers rather than by increasing *m*; however, layered schemes have their own cost per layer, because every layer contributes to the mean optical power, so for a constant optical power, each layer must be allocated less power as the number of layers is increased.

For ease of comparisons, in figure 6, none of the schemes in the figure have diversity combining nor noise cancellation as applied to layered systems by Wang *et al.* [49] and later by Mohammed *et al.* [38], then Wang *et al.* [50] using soft successive interference cancellation. By taking QPSK ACO-OFDM as the reference for SNR cost, Mohammed *et al*.'s fig. 5 can be used by subtracting approximately 5 dB from their optical energy per bit, *E*_b(opt)_/*N*_0_, to obtain the electrical SNR cost of figure 6. Note that their optical energy does not scale as the square-root of electrical SNR as expected for an IM/DD system. This is because the optical power is derived from the expectation value of the clipped OFDM waveform, which equals the standard deviation, *σ*, divided by √(2*π*); this is then normalized for unity optical power, so *σ* = √(2*π*) [51]. The electrical power, including signal and clipping components, is *σ*^2^/2, which thus equals *π* at unity optical power. As the ratio of energies equals the ratio of powers, then *E*_b(opt)_/*N*_0_ = (1/π)*E*_b(elec)_/*N*_0_ [51]. If only useful signals are included in the electrical power, then *E*_b(opt)_/*N*_0_ = (2/*π*)*E*_b(elec)_/*N*_0_, which suggests a 4.8-dB offset at a BER of 10^−3^. Now, using Mohammed's results including diversity combining, we can expect 1.5–2.7-dB reduction in required electrical SNR for single-layer ACO-OFDM (improving with constellation size); for the three-layer ACO-OFDM, the reduction is between 1.25 and 1.5 dB. This improvement due to diversity combining makes layered schemes even more attractive for high spectral efficiencies. Note that their results for DCO-OFDM use a fixed bias of 10 or 13 dB, whereas figure 6 uses optimal biasing for each point, which is far lower for small constellation sizes; for 256-QAM, the optimal bias is 10 dB, so the results converge.

It should be noted that different schemes produce different electrical and optical spectra. This is particularly so for schemes using Flip-OFDM as a basis of each layer. This is because with Flip-OFDM, the spectrum of each subcarrier is spread away from the allocated frequency, where there is a null. This is because the flipping and repetition of the waveform to create the positive then negative frames are functionally identical to repetition, then phase modulation (0, *π*) followed by clipping, so causes a null at the allocated frequency as would binary phase-shift keying. Also, the higher layers only use evens and double-even subcarriers, coinciding with the lower layers. Thus, the overall signal spectrum may be far from flat.

Sun *et al.* [52] have also compared the performance of various schemes, but from the point of view of peak-to-average power ratio (PAPR) and a constraint of 1% of the signals’ duration being out of limit. Although the conclusions were broadly similar, DCO's performance was better than in the above results, though still poorer than three-layer or more schemes. A later paper on the analysis of LACO-OFDM used tone injection to give a further 5-dB reduction in PAPR [53]. Zhou & Zhang [54] have also compared schemes based on the information rate, which assumes unlimited coding to achieve zero error rates, and so is a more fundamental limit. Multi-layer schemes were shown to approach the performance limit of intensity-modulated channels to within 0.07 bits for power-limited optical channels.

## 6. Experimental demonstrations

Since its inception, there have been several experimental demonstrations of single-layer ACO-OFDM for short-haul optical communications and wireless systems, where the low-cost of intensity modulation (a directly modulated laser) combined with a single-photodiode receiver is desirable. Azhar and O'Brien compared several systems, including a DC-biased ACO-OFDM that reintroduced the bias, but kept the odd subcarrier allocation [55]. This helped mitigate baseline wander (effectively the effect of having a high-pass filter) which caused ACO-OFDM to have a penalty despite having a better SNR for a given optical power. Wang *et al.* [56] showed that ACO-OFDM had lower BERs than DCO-OFDM in three-colour links of 10–60 cm for the same data rate. Tahar *et al.* [57] showed that diversity combining provides a 2-dB improvement in 4-QAM ACO-OFDM receiver sensitivity derived from the measured noise variance.

After Tsonev *et al.*'s [30] initial work, there are few experimental demonstrations of layered techniques in wireless systems, though more in short-haul fibre systems where low cost is also desirable. Most recently, Islim and Haas also demonstrated that DMT required 1–2 dB less optical power than DCO-OFDM in 60-cm wireless systems using blue LED and infrared laser; a data rate was not specified though the channel bandwidth was less than 48 MHz [58]. Chen *et al*. [59] have transmitted ADO-OFDM over a 60-cm link using a laser with external modulator and Erbium-doped fibre amplifier using 4-QAM and 16-QAM over a signal bandwidth of nearly 5 GHz. The laser/modulator/amplifier combination is very bulky and costly compared with an LED. Zhang *et al*. [60] have studied error propagation in a seven-layer 7-Gbit s^−1^ laser beam across an optical bench, demonstrating a spectral efficiency of 7 bit s^−1^ Hz^−1^, using bit and power loading that minimized the effect of error propagation.

Very high data rate wireless systems that use slower electrical components can be built using wavelength division multiplexing principles, as are ubiquitous in metro- and long-haul communications systems. These use many electrical transmitters in parallel, each transmitting on an optical wavelength. For example, six lasers, supporting 224 Gbit s^−1^ using a 3-m steered beam, have been demonstrated [61]; this system used a coherent receiver to provide much improved sensitivity over direct detection as the mixing of a local light oscillator and the incoming signal upon photodetection provides gain [62]. More exotically, different channels can be encoded on different modes of a light beam, such as using orbital angular momentum modes [63]. These systems do not fit the mission of this paper, in that we are examining electrical spectral efficiency; however, they should not be forgotten as a way of multiplying the capacity of optical wireless systems, albeit at an exorbitant hardware cost.

Layered techniques have been demonstrated over short-haul (less than 20 km) fibre links, which have a more complex optical channel than wireless links, due to the chromatic dispersion of the fibre. In 2016, Song *et al.* [64] demonstrated a 4.375 Gbit s^−1^ three-layer QPSK system over 19.8 km of fibre using a directly modulated laser and Volterra equalization; a 2-dB improvement in signal quality over DCO-OFDM was measured at the same optical power. Adopting LACO-OFDM does enable the operating range and optimal bias of the laser to be optimized more effectively. Later work using 16-QAM and 64-QAM at 3.5 Gbaud (14/28 Gbit s^−1^) showed a 0.7-dB advantage for 16 QAM, but no advantage for 64-QAM due to error propagation between the layers, exacerbated by the combination of laser chirp and fibre chromatic dispersion [65]; pairwise-coding of good and bad subcarriers within each layer gave a 3.6-dB improvement in signal quality at 20 Gbit s^−1^ mainly due to a reduction in error propagation [66]. Wang *et al*. [67] also demonstrated augmented spectral efficiency discrete multi-tone (ASE-DMT) over fibre, using a single fast Fourier transform (FFT) at the transmitter, as discussed in the next section.

## 7. Efficient digital processing

**Figure 7.**
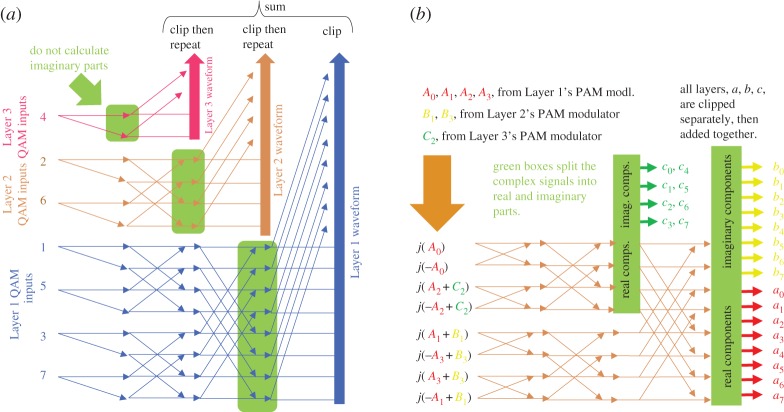
Methods of using a single inverse FFT to generate all layers at the transmitter (and regenerate them at the receiver): (*a*) LACO-OFDM and (*b*) ASE-DMT. (Online version in colour.)

An important issue with most layered methods of improving spectral efficiency is that they require many additional FTs at both the transmitter and receiver; although the transforms for the higher layers can be reduced in length. Wang *et al.* have shown that a single FFT can be modified to generate multiple layers within one transform. The arrangements proposed by Wang *et al*. are shown in figure 7 for LACO [68] and ASE-DMT [67], which is based on layers of PAM. Both techniques rely on extracting signals from within the core of the FFT for two reasons: (i) the waveforms for the higher layers are available from the single transform and (ii) extracting these waveforms is necessary so those subcarriers do not contribute to the final output of the transform. The ASE-DMT method also makes use of the well-known result that one complex-valued-input inverse FT can be used to generate two independent real-valued output streams [69]. This is achieved by applying Hermitian and skew-Hermitian frequency domain inputs across positive and negative frequencies, each producing an independent output after the inverse FFT, as shown in the figure.

## 8. Other improvements

Yang *et al.* [70] have proposed an adaptive scheme where the number of layers is optimized for a given required data rate, for either electrical or optical power constraints and SNRs. Using simulations, they found that the number of layers should be constrained for poor SNRs. Their conclusions support that single-layer ACO-OFDM should be used for poor SNRs, which also means that the SE is poor.

Wang *et al.* [50] have proposed using simplified soft interference cancellation, followed by a second stage of iterative or direct noise clipping, to obtain around 0.6-dB improvement over Mohammed *et al.* [38].

Zhou *et al.* have reduced the PAPR of LACO-OFDM using single-frequency FDM [71], which uses an FT before the transmitter's IFFT, so that the transmitter creates a time domain waveform, with a well-controlled spectrum [72]. A low PAPR is desirable to mitigate transmitter nonlinearities, and also fibre nonlinearities in long-haul systems [73]. Bai *et al.* [74] have proposed real and imaginary separation (RIS) to further reduce PAPR and improve BER in the presence of LED nonlinearity.

In 2013, Zhang & Hanzo proposed a multi-layer modulation scheme [75] similar to superposition coding [76]. Rather than putting different layers on different subcarriers, the layers are superimposed in the time domain with different amplitude weights; for example, superimposing QPSK with a halved amplitude onto QPSK may lead to 16-QAM. The amplitude weights were optimized by a genetic algorithm. Significant gains were achieved over ACO- and DCO-OFDM. Similar algorithms could possibly improve the layered techniques outlined in this paper.

There have been several papers on using multi-layer transmission to optimize the data transmission at different dimming levels. Wang *et al.* [77] have shown that LACO-OFDM in *dimmable* optical OFDM (DO-OFDM) is advantageous over DCO-OFDM at very high and very low levels of illumination. The addition of pulse-width modulation gives many possibilities as discussed in a recent paper by Li *et al.* [78].

## 9. Musical perception

**Figure 8.**
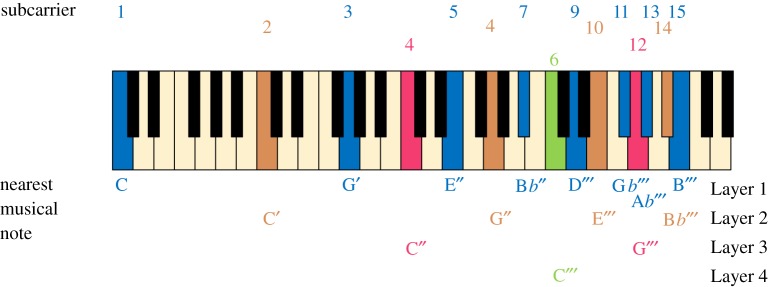
Musical representation of LACO-OFDM as a series of chords at octave intervals, each conveying a layer. The chords consist of odd harmonics of the fundamental note, C. (Online version in colour.)

The frequency allocation of LACO-OFDM subcarriers can be mapped onto a musical scale, as illustrated in figure 8. Each layer has a series of notes that are odd multiples in frequency (i.e. harmonics) of a fundamental tone, forming a chord. The higher layers start their chords at octaves above the previous layer. The processing of LACO-OFDM starts with the lowest chord and cancels it, and its distortion products, to reveal the next chord up.

It is interesting to speculate whether the brain uses similar processing to separate out musical instruments in orchestral and other music, particularly rock music with heavily distorted instruments. If the brain could first detect the lowest layers (such as a bass guitar), it would be able to recreate the harmonic series of each note, so could cancel out the higher harmonics, to reveal higher pitch instruments. This process could be repeated through the mid-range instruments, to the highest pitch instruments or voice. In rock music, each instrument is usually quite distorted, but importantly the distortion is created in a separate ‘guitar’ amplifier for each instrument, so is harmonically related to each instrument, so may enable the brain to decipher the music. However, if all instruments are played through a single distorting amplifier, then the sound becomes awful; this was the experience of many teenage ‘garage’ bands who could only afford a single amplifier; it may also be the reason why live music generally sounds better than recorded music.

## 10. Conclusion

The quest for power efficiency in intensity-modulated optical OFDM systems resulted in strongly asymmetrically clipped waveforms that did not require additional DC-bias; unfortunately, these halved the electrical spectral efficiency because many of the subcarrier frequency slots became polluted by strong clipping distortion. The invention of layered techniques, in which the clipping distortion is mostly cancelled by estimating the data on at least one unpolluted layer, then subtracting this to reveal the wanted signals on deeper layers, has helped regain the spectral efficiency. Importantly, layered systems using QAM theoretically require lower SNRs at the receiver than conventional systems that use DC-bias or PAM that requires no bias. In addition, diversity techniques can be used to gain further advantage because the clipping distortion also contains information. Thus, layered systems should be adopted to maximize the performance of intensity-modulated links.

A cost of layered systems is increased signal processing complexity at the transmitter and receiver, to generate and then ‘peel back’ the layers, respectively. Some advances have been made using ‘middle-out’ fast FTs, which are able to generate multiple layers with a single transform. These reduce the processing at the transmitter, and also at the receiver because the receiver requires ‘transmitter processing’ to regenerate the layers during the cancellation process. Obviously, there is room for improvement in the receiver processing (except for spectral and energy efficient-OFDM)––to reduce the number of transforms. There is also much work to be done demonstrating the advantages of layered techniques in real systems with imperfections (nonlinearities and memory) in the components, particularly in modulated lasers.

## Data accessibility

This article has no additional data.
